# Patient, prisoner or person?

**DOI:** 10.1186/1477-7517-3-23

**Published:** 2006-08-07

**Authors:** Dan Small

**Affiliations:** 1 Director PHS Community Services Society, 20 West Hastings Street, Vancouver, BC, V6B 1G6, Canada; 2 Research Associate, Department of Anthropology and Sociology, University of British Columbia, Vancouver, BC, V6T 1Z1, Canada

## Abstract

Case studies provide rich descriptions of significant vignettes that highlight atypical systemic or clinical problems and identify potentially important research questions. The case study presented by Venters, Razvi, Tobia and Drucker (2006) describes an unfortunate set of events pertaining to an individual's experience as they were failed by s several systems all at once and neglected for having had experience with an addiction. This commentary provides some remarks on the case study with respect to differing institutional narratives as they pertain to lived experience in the context of everyday life. It is suggested that, in the special case of addiction, the mistreatment of the subject of the case study, Mr. Ortiz, is not an exception to the norm, but the norm itself for people living with addictions and their families.

The case study presented by the Venters, Razvi, Tobia and Drucker [1] in this volume portrays, all at once, a legal failing, a medical misadventure and personal tragedy. To the various systems, Mr. Ortiz is part patient, part prisoner and part person. The criminal justice plot line begins with a prisoner on the road to rehabilitation. Mr. Ortiz's lack of access to adequate healthcare (i.e. testing and treatment for HCV) indicates how his personhood was diminished within this setting. A public health narrative would have, presumably, offered a different beginning, middle and end point where the central character would have been seen as a site for illness, disease and treatment. Rich descriptions of individual experience like this one help us to identify gaps in service and to define relevant research questions. But they do something more; they identify our professional shortcomings and the way that systems, of which we are all a part, often fail the people with the most needs.

Differing institutional narratives, like the ones described in the present case study, can lead to dramatically different outcomes for patients and persons. For example, in a comparison between two hospitals, it was discovered that in one hospital, physicians limited at least one type of life prolonging equipment for 1 in 4 (25%) patients discharged from the ICU whereas doctors in another hospital imposed these limitations in only 1 in 7 (14%) patients [2]. Interviews revealed that there were not any significant differences in the medical or ethical orientations of the physicians in the two hospitals. In fact, the key decisions about end of life care were imposed on the physicians by the administrators in the respective hospitals based on their differing analyses of the risk of lawsuits following the removal of patients from respirators.

Notwithstanding, these systems are still, at their very heart, comprised of human beings, professionals, with the capacity to reflect upon institutional arrangements and to put their client's narrative, rather than the institutional narrative, at the centre of care. The only way to bring the differing narratives of various systems (e.g health, criminal justice, legal, personal) together so that they share a common plot line is by encouraging professional and institutional reflexivity (self awareness within systems). This self-awareness begins by encouraging professional understanding about the way institutions typically fit people to systems rather than systems to people. By promoting understanding of institutional narratives, we can work towards systemic accountability and, ultimately, a more person-centred design.

Why person-centred? For the reason that the experience of illness, like HCV, doesn't really occur in the criminal justice system, courtroom or clinics.

It occurs in a deeply more personal landscape; the lifeworld. It is Mr. Ortiz who will ultimately have to negotiate the impact of these fateful moments on his personhood. By adopting a reflective approach, where we put the experience of those most affected by systems in the centre of our narrative, we can move beyond the sad story at the heart of this case to ensure that this is not a normal experience, but an exception.
